# Self‐Templating and In Situ Assembly of a Cubic Cluster‐of‐Clusters Architecture Based on a {Mo_24_Fe_12_} Inorganic Macrocycle

**DOI:** 10.1002/anie.201603298

**Published:** 2016-06-30

**Authors:** Weimin Xuan, Andrew J. Surman, Qi Zheng, De‐Liang Long, Leroy Cronin

**Affiliations:** ^1^ WestCHEM, School of ChemistryThe University of GlasgowGlasgowG12 8QQUK

**Keywords:** anion templating, host–guest chemistry, macrocycles, polyoxometalates, supramolecular cages

## Abstract

Engineering self‐templating inorganic architectures is critical for the development of bottom‐up approaches to nanoscience, but systems with a hierarchy of templates are elusive. Herein we describe that the cluster‐anion‐templated (CAT) assembly of a {CAT}⊂{Mo_24_Fe_12_} macrocycle forms a giant ca. 220 nm^3^ unit cell containing 16 macrocycles clustered into eight face‐shared tetrahedral cluster‐of‐clusters assemblies. We show that {CAT}⊂{Mo_24_Fe_12_} with different CATs gives the compounds **1**–**4** for CAT=Anderson {FeMo_6_} (**1**), Keggin {PMo_12_} (**2**), Dawson {P_2_W_18_} (**3**), and {Mo_12_O_36_(HPO_3_)_2_} (**4**) polyoxometalates. “Template‐free” assembly can be achieved, whereby the macrocycle components can also form a template in situ allowing template to macrocycle to superstructure formation and the ability to exchange the templates. Furthermore, the transformation of template clusters within the inorganic macrocycle {Mo_24_Fe_12_} allows the self‐generation of an uncapped {Mo_12_O_36_(HPO_3_)_2_} in compound **4**.

Anions are fundamentally important in biological systems,^1^ and biology has evolved receptors to recognize, detect, and transport anions.^2^ In this sense, nature builds complex supramolecular assemblies around the “anion templates” to perform functions.^3^ As such we were interested in using these principles in all‐inorganic systems based upon polyoxometalate (POM) clusters to see if dynamic self‐assembling systems can be produced. POMs are a unique class of discrete metal oxides with a wide variety of structures and properties.^4^, ^5^, ^6^ Their intrinsically anionic nature, variable charge, size, and redox activity make them ideal candidates for the functional anion‐driven assembly of architectures.^7^, ^8^ For instance, it has been shown that the intrinsic formation of {Mo_36_} upon acidification of molybdate allows the self‐assembly of the {Mo_154_} giant Mo‐blue macrocycle,^9^ opening the way for the formation of the family of gigantic polyoxomoblybdates ranging in nuclearities from 36 to 368. In this respect it is increasingly thought that the host–guest [{Mo_36_}⊂{Mo_150_}] system is the gateway to these architectures,^10^ being the key intermediate that directs the self‐assembly of an otherwise, impossible or highly improbably set of structures.^9^, ^10^ Similarly the formation of [(cluster anion)^*n*−^⊂[Mo_24_Fe_12_(EDTA)_12_O_72_]^(12+*n*)−^ is an example of a macrocycle8a that can bind large anions. In this respect we hypothesized that nanoscale macrocyclic systems could exploit the template‐driven assembly to form larger architectures, and also self‐select or self‐synthesize new templates that are so far not known, see Scheme 1.

**Scheme 1:**
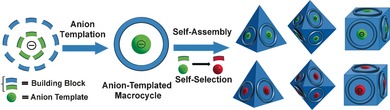
Schematic representation of the dynamic assembly of anion‐templated macrocycles as a function of the anion, and then the cluster‐of‐clusters assembly. The system can re‐select new anions from the available library in solution via an in situ transformation process.

By exploring the dynamic assembly of an inorganic‐macrocycle system, based upon the inorganic ring [Mo_24_Fe_12_(EDTA)_12_O_72_]^12−^, we show that the assembly can be directed by the same building‐block mixture that forms the macrocycle, see Figure 1, and that the system self‐selects a cluster‐anion template (CAT) that stabilizes the ring. In confirming our hypothesis we discovered four POM‐templated macrocycles:

**Figure 1:**
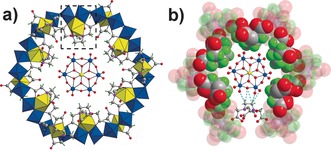
a) View of the molecular structure of the macrocycle (polyhedron model) and template (ball‐and‐stick model) found in **1**–**4**. The actual structure is the {CAT}⊂{Mo_24_Fe_12_} found in compound **1** where the {CAT}={FeMo_6_} Anderson cluster. The Mo_2_Fe(EDTA) unit is highlighted by the dashed box. Fe yellow; Mo blue; O red; C gray; N pink; H bright green; b) Representation of hydrogen bonds (turquoise dotted lines) formed between one sixth of the Anderson guest and one EDTA ligand.

Na_15_[(H_6_FeMo_6_O_24_)⊂Mo_24_Fe_12_(EDTA)_12_O_72_]**⋅**80 H_2_O (**1**)

≡ Na[**1 a**]**⋅**80 H_2_O

Na_15_[(PMo_12_O_40_)⊂Mo_24_Fe_12_(EDTA)_12_O_72_]⋅90 H_2_O (**2**)

≡ Na_15_[**2 a**]**⋅**90 H_2_O

Na_18_[(P_2_W_18_O_62_)⊂Mo_24_Fe_12_(EDTA)_12_O_72_]**⋅**100 H_2_O, (**3**)

≡ Na_18_[**3 a**]**⋅**100 H_2_O

Na_16_[(Mo_12_O_36_(HPO_3_)_2_(H_2_O)_6_)⊂Mo_24_Fe_12_(EDTA)_12_O_72_]⋅85 H_2_O (**4**) ≡ Na_16_[**4 a**]**⋅**85 H_2_O

All the compounds were characterized crystallographically and using extensive analytical techniques (see Supporting Information) and all four compounds crystallize in the same F*d*‐3 lattice with cell axes of 61.2 Å and unit cell volume of over 222 000 Å^3^.

In our initial experiments we set out to deduce if an “intrinsic” template for the {Mo_24_Fe_12_(EDTA)_12_} existed or if an external template had to be added to either select or assemble the building blocks in solution. This was done by mixing the correct stoichiometry of Na_2_MoO_4_, FeCl_3_ **⋅**6 H_2_O, and Na_2_EDTA (in a ratio of 2:1:1) in aqueous solution aiming to produce the empty form of the {Mo_24_Fe_12_(EDTA)_12_} macrocycle.8a Crucially no pre‐formed anion templates, such as the {M_12_} Keggin or {M_18_} Dawson, were added, yet to our surprise high‐quality crystals of **1** were obtained in two weeks. Single‐crystal X‐ray structure analysis of **1** reveals a host–guest structure **1 a** in which an Anderson‐type anion [FeMo_6_O_18_(OH)_6_]^3−^ ({FeMo_6_}) is entrapped by the cyclic host {Mo_24_Fe_12_(EDTA)_12_} (Figure 1 a). The host features a twisted ring shape that is composed of 12 Mo_2_Fe(EDTA) repeat units. In each unit, the Fe site adopts seven‐coordinate pentagonal bipyramidal geometry enclosed by one EDTA ligand and one μ_3_‐O while the two MoO_6_ octahedra are bound with the Fe center via the μ_3_‐O and two carboxylate O atoms from EDTA. Bond valence sum (BVS) calculations indicate all the Mo and Fe centers adopt +6 and +3 oxidation state, respectively.^11^

The Anderson template resides exactly at the center of macrocycle, with a *C*_3_ axis passing through the central Fe atom, (Figure 1), and is anchored in place by a large number of C−H⋅⋅⋅O hydrogen‐bonds with the coordinated EDTA ligands grafted to the inner ring of the macrocycle (see Figure 1). Apart from the aesthetic beauty of the molecular structure, **1 a** exhibits a remarkable supramolecular architecture and gigantic protein‐sized unit cell (cell axis is 6 nm long) with a volume of approximately 229 nm^3^. Each unit cell contains sixteen inorganic macrocycles with templates and these are arranged into four supramolecular tetrahedral cages whereby each of the four faces of the tetrahedron are capped by a {CAT}⊂{Mo_24_Fe_12_} via hydrogen bonds formed between adjacent macrocycles and the internal void volume of the supramolecular tetrahedron is approximately 7238 Å^3^; in fact a crystallographic analysis indicates that about 64 % of the unit‐cell volume is occupied by solvent and counterions. The adjacent tetrahedrons stack on each other in a face‐shared mode, that is, one tetrahedron is surrounded by four other tetrahedrons by sharing all its four faces (Figures 2). In this respect, one tetrahedron could be simplified as a 4‐connected node (Figure 2 c) that link with each other in a tetrahedral arrangement, leading to a 3D Cubic F network (Figure 2 d). Compound **1** is a rare example of a POMs‐based macrocycle with a mesoporous cage‐like void built inside that has been crystallographically characterized.

**Figure 2:**
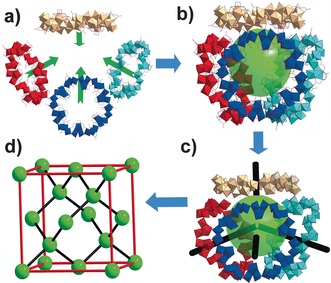
a) Schematic representation of the construction of supramolecular tetrahedron. The four **1 a** macrocycles are presented in different colors and Anderson templates are omitted for clarity; b) View of the supramolecular tetrahedron with a green ball to highlight the void space; c) 4‐connected node simplified from one tetrahedron; d) Cubic F 3D network constructed from connection of 4‐connected nodes (green spheres).

Given the existence of an intrinsic or “natural” template, we wished to see if we could induce the formation of other templates. Based on the stoichiometry of previously reported [(SiMo_12_O_40_)⊂Mo_24_Fe_12_(edta)_12_O_72_]^16−^ where the ratio of Mo, Fe, and EDTA is 3:1:1 we adapted the synthetic conditions to mix Na_2_MoO_4_, FeCl_3_ **⋅**6 H_2_O, and Na_2_EDTA in this ratio together with 1/12 equivalent of Na_3_PO_4_. The phosphate source was used to induce the formation of phosphorous‐centered Keggin‐type template [PMo_12_O_40_]^3−^ ({PMo_12_}), and crystals of **2** were obtained in two weeks. Consistent with compound **1**, the single‐crystal X‐ray structure analysis of **2** reveals a host–guest structure **2 a** in which a Keggin‐type anion {PMo_12_} is bound by the cyclic host {Mo_24_Fe_12_(EDTA)_12_}. Compound **2 a** is isostructural to **1 a** but with the {PMo_12_} located at the center of the macrocycle. Since {PMo_12_} is ambiguously recognized from the crystal structure, it is evident that the Keggin anion could be produced in situ as well, but to check this we also added {PMo_12_} directly to the reaction mixture which also resulted in the isolation of **2**.8a Similarly, the addition of the {P_2_W_18_} Dawson anion yielded **3**. It should be noted that the molecular structures of **2** and **3** are similar to the previously reported [(SiMo_12_O_40_)⊂Mo_24_Fe_12_(EDTA)_12_O_72_]^16−^ and [(P_2_W_18_O_62_)⊂Mo_24_Fe_12_(EDTA)_12_O_72_]^18−^, albeit with different counterions/heteroatoms and different supramolecular structures as shown in Figure S4. The improved resolution of crystal data for **3** compared to **1** and **2**, allowed six crystallographically equivalent sodium ions to be found residing between EDTA ligands and the {P_2_W_18_} guest in **3 a** (Figure S4). The sodium ions adopt an octahedral coordination geometry defined by two oxygen atoms from two EDTA ligands, one terminal oxygen atom from belt layer of {P_2_W_18_}, and three water ligands. The anionic template and cyclic host are thus connected by sodium ions, which act as a charge buffer to reduce repulsive electrostatic force between two negatively charged species. This buffering has already been seen for gigantic {Mo_36_}⊂{Mo_150_} cluster in which sodium ions distribute between anionic {Mo_36_} template and {Mo_150_} host.^10^, ^12^ In addition to sodium ions, multiple hydrogen bonds are detected between the host and anionic templates which also contribute to stabilize the host–guest assemblies in **1**–**3**. All the three anionic guests make full use of surface oxygen atoms (both terminal and bridging) to achieve optimal and maximum hydrogen bonding. Whist {PMo_12_} and {P_2_W_18_} utilize oxygen atoms either from belt and cap areas to form hydrogen bonds within and around the cavity, the planar {FeMo_6_} could only utilize oxygen atoms located in the same plane (Figure S5). Fewer hydrogen bonds are generated for the Anderson anion and this explains why the addition of {PMo_12_} and {P_2_W_18_} suppresses the in situ formation of {FeMo_6_}, that is, both {PMo_12_} and {P_2_W_18_} guests are trapped preferentially in the cyclic host.

To explore the preference of macrocycle for the {PMo_12_} and {P_2_W_18_} guests rather than the {FeMo_6_}, we performed template‐exchange by using a tenfold excess of the {PMo_12_} or {P_2_W_18_} in an aqueous solution of **1** which was dissolved by heating. The template‐exchanged samples were identified as **2** and **3** by single‐crystal X‐ray diffraction and elemental analysis, indicating successful replacement of {FeMo_6_} by {PMo_12_} and {P_2_W_18_}. Furthermore, the exchange process could also be monitored in situ by ^31^P NMR spectroscopy. In this experiment one equivalent of compound **1** as a powder was divided into three parts and added sequentially as to a solution of one equivalent of {P_2_W_18_} in D_2_O. After each addition, ^31^P NMR measurements showed a decrease in the signal related to {P_2_W_18_} species in solution, indicating that {P_2_W_18_} was included into {Mo_24_Fe_12_} whose paramagnetic shell shielded the encapsulated guest (Figure S15). We also checked the template exchange of {PMo_12_} with {P_2_W_18_} in a similar way, and came to the same conclusion as the previous work,8a that is, {P_2_W_18_} couldn't replace {PMo_12_}, and visa versa. Therefore, the sequence of binding ability to {Mo_24_Fe_12_} is {P_2_W_18_}≈{PMo_12_}>{FeMo_6_}, which is consistent with the number of hydrogen bonds (Figure S5) and the degree of close packing between the CAT and {Mo_24_Fe_12_} (Figure S7).

The common structural feature of {FeMo_6_}, {PMo_12_}, and {P_2_W_18_} lies in the equatorial belt layer consisting of M_6_(XO_*n*_) (M=Mo or W, X=Fe or P, and *n*=6 or 4). The size and geometry of this M_6_ belt perfectly fits the diameter and symmetry of macrocycle, whist the oxygen atoms on the surface are receptors for hydrogen bonds or potential oxo‐ligands for sodium ions, providing stability to the host–guest aggregate. This indicates that the macrocycle selectively captures anionic clusters with similar transverse profile as templates, in addition to the in situ formation of {FeMo_6_} and {PMo_12_} within the macrocycle. With this in mind, we started to explore the possibility of using the macrocycle as a confined reaction vessel whereby unstable “anionic templates” of the right size may be produced in situ and stabilized within the cavity of the host. For this purpose, we selected HPO_3_^2−^ as the hetero‐anion for the construction of anionic template because of its unique trigonal pyramid configuration.^13^ The synthetic procedure is similar to **2** but using H_3_PO_3_ instead of Na_3_PO_4_. Crystals of **4** were obtained within two weeks and structural analysis of **4** reveals a novel host–guest structure **4 a** where an uncapped Dawson‐type anion [Mo_12_O_36_(HPO_3_)_2_(H_2_O)_6_]^4−^ ({Mo_12_(HPO_3_)_2_}) is wrapped by the cyclic host {Mo_24_Fe_12_(EDTA)_12_} (Figure 3 a). Compound **4 a** is isostructural to **1 a**, **2 a**, and **3 a** but with {Mo_12_(HPO_3_)_2_} in the center. Multiple hydrogen bonds could also be detected between {Mo_12_(HPO_3_)_2_} and EDTA ligands (Figure S6).

**Figure 3:**
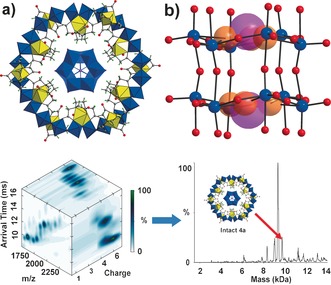
Top: a) View of the molecular structure of **4 a**. Color code is the same as Figure 1; b) View of the embedded {Mo_12_(HPO_3_)_2_}. The [HPO_3_]^2−^ anion is highlighted in space filling representation and H atoms are omitted for clarity. Mo blue; P pink; O red and orange. Bottom: IMS‐MS data and charge assignments (left) and deconvoluted neutral mass spectrum (right) of a solution of **4**.

The structure of anionic template {Mo_12_(HPO_3_)_2_} in **4 a** comprises two {Mo_6_(HPO_3_)} layers connected by six oxygen atoms. Each layer is composed of six MoO_6_ octahedra linked together by the central [HPO_3_]^2−^ in a cyclic form via edge and corner‐shared modes (Figure 3 b). The interlayer Mo⋅⋅⋅Mo distance is 4.0299(3) Å and the six Mo atoms in each layer adopt quasi‐chair conformation (Figure S6), and analysis indicates all the Mo centers adopt +6 oxidation state (Table S2). Overall, the {Mo_12_(HPO_3_)_2_} cluster can be considered as a hexa‐lacunary Dawson cluster, and such a compound is not stable in the aqueous phase although a similar organic‐soluble species [Mo^V^_4_Mo^VI^_8_O_30_(Mo^VI^O_4_)_2_(OAc)_6_]^2−^ has been reported.^14^ Therefore, {Mo_12_(HPO_3_)_2_} represents the first Dawson‐type polyoxomolybdate fragment to be observed. The species corresponding to the mass of the intact host–guest complex could even be detected directly in solution using ion‐mobility electrospray mass spectrometry; the ion‐mobility data can be de‐convoluted giving a neutral mass spectrum (see Figure 3).

The successful synthesis of compound **4** validates our idea to use the macrocycle as a confined reaction container. The underlying principle is that the formation of macrocycle is dependent on the anionic template, and the macrocycle itself will select the most suitable template that can match the size and symmetry of a macrocycle based on the ingredients (hetero‐anions) we feed. On the basis of what was discussed above, we postulate that the overall mechanism underpinning the formation of the {Mo_24_Fe_12_(EDTA)_12_} family involves anionic POMs clusters as structure‐directing templates which can be either added exteriorly or form in situ (Scheme 2). The process could be described by the reaction of Na_2_MoO_4_, FeCl_3_, and Na_2_EDTA, whereby the {FeMo_6_} Anderson anion will be generated in situ to drive the self‐assembly of **1**. If the pre‐formed Keggin {PMo_12_} and Dawson {P_2_W_18_} anions are added into the system, the initiation of {FeMo_6_} is prevented and **2** and **3** will be built via the templating of {PMo_12_} and {P_2_W_18_}. Compounds **2** and **3** could also be obtained by template‐exchange of {FeMo_6_} in **1** with {PMo_12_} and {P_2_W_18_}. Instead of addition, the {PMo_12_} anion could also form in situ as a template that is built from the hetero‐anion PO_4_^3−^. In a similar way, {Mo_12_(HPO_3_)_2_} is produced in situ within the cavity of **4** by the introduction of HPO_3_^2−^.

**Scheme 2:**
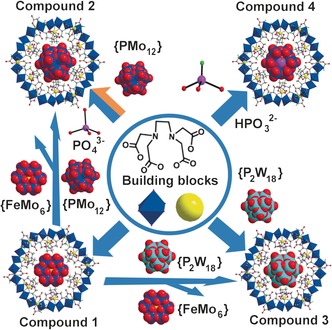
The anion‐templated self‐assembly of macrocycles **1**–**4** and transformation from **1** to **2** and **3** by template exchange.

In summary, we present a series of anion‐templated macrocycles **1**–**4** featuring a soft hybrid network built from porous supramolecular tetrahedrons. The stability of these host–guest assemblies are demonstrated by the IMS spectrometry. The successful construction of **1** unveils the “intrinsic” minimal template in this system is Anderson anion {FeMo_6_} formed in situ, which could be exchanged by {PMo_12_} and {P_2_W_18_}, leading to the formation of **2** and **3**. The driving force for the template exchange originates from the greater number of hydrogen bonds formed between the cyclic host and {PMo_12_}/{P_2_W_18_} in comparison with {FeMo_6_} as shown crystallographically. Direct addition of pre‐formed {PMo_12_} and {P_2_W_18_} templates into the reaction system could also result in **2** and **3**. Moreover, the anionic {PMo_12_} template can be also generated in situ to direct the formation of **2**. Based on the discovery of **1**–**3** and the facile transformation from **1** to **2** and **3** through template exchange, the formation mechanism for these host–guest aggregates illustrates the self‐assembly is essentially template dependent. Finally, the concept of using a macrocycle as a confined reaction vessel is verified by the discovery of **4** of which the uncapped Dawson {Mo_12_(HPO_3_)_2_} cluster is generated in situ within the cavity of the {Mo_24_Fe_12_(EDTA)_12_} macrocycle. In the future, we will further extend the utility of the {Mo_24_Fe_12_(EDTA)_12_} macrocycle as confined reaction vessel to discover novel anionic clusters.

## Supporting information

As a service to our authors and readers, this journal provides supporting information supplied by the authors. Such materials are peer reviewed and may be re‐organized for online delivery, but are not copy‐edited or typeset. Technical support issues arising from supporting information (other than missing files) should be addressed to the authors.

Supplementary
