# Supplementary material for: Self‐Templating and In Situ Assembly of a Cubic Cluster‐of‐Clusters Architecture Based on a {Mo24Fe12} Inorganic Macrocycle
Source: Angew Chem Int Ed Engl. 2016 Jun 30;55(41):12703–7. doi: 10.1002/anie.201603298 (PMC5396355; doi:10.1002/anie.201603298)
Supplement: Supplementary file 1 — Supplementary [file ANIE-55-12703-s001.pdf]

Angewandte Chemie
Eine Zeitschrift der Gesellschaft Deutscher Chemiker

Supporting Information

Self-Templating and In Situ Assembly of a Cubic Cluster-of-Clusters Architecture Based on a $\{Mo_{24}Fe_{12}\}$ Inorganic Macrocycle

*Weimin Xuan, Andrew J. Surman, Qi Zheng, De-Liang Long, and Leroy Cronin**

anie_201603298_sm_miscellaneous_information.pdf

Table of Contents

1. Materials S2

2. Instrumentation S2

3. Synthetic procedure of **1-4** S3

4. Crystallographic data and crystal structures of **1-4** S5

5. TGA of **1-4** S10

6. PXRD spectra of **1** S12

7. ESI-Ion Mobility Mass Spectrometry data of **1-4** S12

8. Transformation from **1** to **2** and **3** by template exchange and ^{31}P NMR study of template exchange S14

9. BET plot and N_2 adsorption of **1** S15

10. References S17

1. Materials

Reagent-grade chemicals were obtained from Aldrich Chemical Company Ltd. and Alfa Aesar, and used without further purification. $Na_6P_2W_{18}O_{62}\cdot14H_2O$ was prepared according to literature.[1]

2. Instrumentation

Crystallography: Suitable single crystal was selected and mounted onto a rubber loop using Fomblin oil. X-ray diffraction intensity data a Bruker Apex 2 CCD diffractometer (λ (MoK_α) = 0.7107 Å) equipped with a microfocus x-ray source (50kV, 30w). Data collection and reduction were performed using the Apex2 software package and structure solution, and refinement were carried out using SHELXS-97[2] and SHELXL-97[3] using WinGX.[4] Corrections for incident and diffracted beam absorption effects were applied using empirical absorption correction.[5] All the non-hydrogen atoms (including those disordered) were anisotropically refined. CCDC 1471467−1471470 contain the supplementary crystallographic data for compound **1-4**. These data can be obtained free of charge via www.ccdc.cam.ac.uk/data_request/cif, or by emailing data_request@ccdc.cam.ac.uk, or by contacting The Cambridge Crystallographic Data Centre, 12 Union Road, Cambridge CB2 1EZ, UK; fax: +44 1223 336033.

ESI-Ion Mobility Mass Spectrometry: The spectra were acquired on a Waters Synapt G2 HDMS instrument in Sensitivity mode (except where stated), with samples infused into the standard ESI source at 5 µl/min using a Harvard syringe pump.

^{31}P Nuclear Magnetic Resonance Spectroscopy: ^{31}P NMR spectroscopy was recorded on a Bruker DPX 500 spectrometer. All samples were prepared by dissolving the clusters in D_2O.

Fourier-transform infrared (FT-IR) spectroscopy: The samples were prepared as a KBr pellet and the FT-IR spectrum was collected in transmission mode in the range of 400-4000 cm^{-1} using a JASCO FT-IR 4100 spectrometer. Wavenumbers are given in cm^{-1}. Intensities are denoted as w = weak, m = medium, s = strong, br = broad, sh = sharp.

Element Analyses: Element analyses for Mo, W, Fe, P and Na were performed on a Leeman inductivity-coupled plasma (ICP) spectrometer while C, N and H content were determined by the microanalysis services within the Department of Chemistry, University of Glasgow using an EA 1110 CHNS, CE-440 Elemental Analyzer.

Thermogravimetric Analysis (TGA): Thermogravimetric analysis was performed on a TA Instruments Q 500 Thermogravimetric Analyzer under nitrogen flow at a typical heating rate of 10°C min^{-1}.

Powder X-ray Diffraction (PXRD): Powder XRD was recorded on a Bruker D8X diffractometer equipped with monochromatized Cu-Kα (λ = 1.5418 Å) radiation in the range of $2 \leq 2\theta \leq 50$, with a scanning rate of 4° s^{-1}

3. Synthetic procedure of 1-4

1: $Na_{15}[(FeMo_6O_{24}H_6)\subset Mo_{24}Fe_{12}(EDTA)_{12}O_{72}]\cdot 80H_2O$, Na_{15}**[1a]**$\cdot 80H_2O$, M.W.: 10391.7

Ethylenediaminetetraacetic acid disodium salt dihydrate (0.093 g, 0.25 mmol) and $FeCl_3\cdot 6H_2O$ (0.067 g, 0.25 mmol) were added to a solution of $Na_2MoO_4\cdot 2H_2O$ (0.120 g, 0.5 mmol) in water (8 mL). The mixture was vigorously stirred for 20 min until a clear red solution is formed. The solution was then acidified with 1 M HCl (0.3 mL) to pH 2.4. After heating at 90 °C for 30 min, the clear yellow solution was allowed to cool to room temperature and kept in an open 50 mL Erlenmeyer flask undisturbed for 2 weeks. The resulting yellow diamond-like crystals were collected and dried in air (yield 58 mg, 28 % based on Fe). Due to the photosensitivity of compound **1**, the dried sample was stored in dark. Elemental analysis, calc.: C, 13.87%; H, 3.00 %; N, 3.23%; Na, 3.31 %; Fe, 6.98 %; Mo, 27.70 %; found: C, 16.4%; H, 3.4%; N, 3.5%; Na, 3.06 %; Fe, 6.77%; Mo, 28.0 %. IR (KBr pellet, 3000–400 cm^{-1}): 2957(w), 2923(w), 1638(s), 1440(sh), 1384(s), 1327(m), 1267(sh), 1215(sh), 1109(sh), 1027(w), 1004(w), 950(s), 930(sh), 845(w), 789(m), 735(m), 599(s, br), 514(sh), 422(w).

2: $Na_{15}[(PMo_{12}O_{40})\subset Mo_{24}Fe_{12}(EDTA)_{12}O_{72}]\cdot 90H_2O$, Na_{15}**[2a]**$\cdot 90H_2O$, M.W.:11372.35

Method 1: Ethylenediaminetetraacetic acid disodium salt dihydrate (0.093 g, 0.25 mmol) and $FeCl_3\cdot 6H_2O$ (0.067 g, 0.25 mmol) were added to a solution of $Na_2MoO_4\cdot 2H_2O$ (0.120 g, 0.5 mmol) in water (8 mL). The mixture was vigorously stirred for 20 min until a clear red solution is formed. The solution was then acidified with 1 M HCl (0.3 mL) to pH 2.4, and $H_3PMo_{12}O_{40}\cdot 12H_2O$ (0.040 g, 0.02mmol) then added. After heating at 90 °C for 30 min, the clear yellow solution was allowed to cool to room temperature and kept in an open 50 mL Erlenmeyer flask undisturbed for 2 weeks. The resulting yellow diamond-like crystals were collected and dried in air (yield 84 mg, 35 % based on Fe). Due to the photosensitivity of compound **2**, the dried sample was stored in dark. Elemental analysis, calc.: C, 12.67%; H, 2.87%; N, 2.95%; Na, 3.03%; P, 0.27%; Fe, 5.89%; Mo, 30.37%; found: C, 12.43%; H, 2.14%; N, 2.88%; Na, 3.17%; P, 0.25%; Fe, 5.91%; Mo, 29.30%. IR (KBr pellet,3000–400cm^{-1}): 2958(w), 2924(w), 1640(s), 1441(sh),1384(s), 1327(sh), 1268(sh), 1214(sh), 1109(sh), 1055(m), 1027(w), 1004(w), 950(s), 930(s), 876(m), 801(s), 735(m), 599(s, br), 514(sh), 427(w).

Method 2: Ethylenediaminetetraacetic acid disodium salt dihydrate (0.093 g, 0.25 mmol) and $FeCl_3\cdot 6H_2O$ (0.067 g, 0.25 mmol) were added to a solution of $Na_2MoO_4\cdot 2H_2O$ (0.180 g, 0.75 mmol) in water (8 mL). The mixture was vigorously stirred for 20 min until a clear red solution is formed. The solution was then acidified with 1 M HCl (0.3 mL) to pH 2.4, and Na_3PO_4 (3.5 mg, 0.02mmol) then added. After heating at 90 °C for 30 min, the clear yellow solution was allowed to cool to room temperature and kept in an open 50 mL Erlenmeyer flask undisturbed for 2 weeks. The resulting yellow diamond-like crystals were collected and dried in air (yield 76 mg, 31 % based on Fe). Due to the photosensitivity of compound **2**, the dried sample was stored in dark. Elemental analysis, calc.: C, 12.67%; H, 2.87%; N, 2.95%; Na, 3.03%; P, 0.27%; Fe, 5.89%; Mo, 30.37%; found: C, 12.43%; H, 2.14%; N, 2.88%; Na, 3.17%; P, 0.25%; Fe, 5.91%; Mo, 29.30%. IR (KBr pellet,3000–400cm^{-1}):

2958(w), 2924(w), 1640(s), 1441(sh),1384(s), 1327(sh), 1268(sh), 1214(sh), 1109(sh), 1055(m), 1027(w), 1004(w), 950(s), 930(s), 876(m), 801(s), 735(m), 599(s, br), 514(sh), 427(w).

3: $Na_{18}[(P_2W_{18}O_{62})\subset Mo_{24}Fe_{12}(EDTA)_{12}O_{72}]\cdot 100H_2O$, Na_{18}**[3a]**$\cdot 100H_2O$, M.W.: 14162.04

Ethylenediaminetetraacetic acid disodium salt dihydrate (0.093 g, 0.25 mmol) and $FeCl_3\cdot 6H_2O$ (0.067 g, 0.25 mmol) were added to a solution of $Na_2MoO_4\cdot 2H_2O$ (0.120 g, 0.5 mmol) in water (8 mL). The mixture was vigorously stirred for 20 min until a clear red solution is formed. The solution was then acidified with 1 M HCl (0.3 mL) to pH 2.4, and $Na_6P_2W_{18}O_{62}\cdot 14H_2O$ (0.090 g, 0.02 mmol) then added. After heating at 90 °C for 30 min, the clear yellow solution was allowed to cool to room temperature and kept in an open 50 mL Erlenmeyer flask undisturbed for 2 weeks. The resulting yellow diamond-like crystals were collected and dried in air (yield 97 mg, 33 % based on Fe). Due to the photosensitivity of compound **3**, the dried sample was stored in dark. Elemental analysis, calc.: C, 10.17%; H, 2.44%; N, 2.37%; Na, 2.92%; P, 0.42%; Fe, 4.73%; Mo, 16.26%; W, 23.36%; found: C, 9.82%; H, 1.69%; N, 2.53%; Na, 2.53%; P, 0.41%; Fe, 4.61%; Mo, 16.10%; W, 21.90%. IR (KBr pellet,3000–400cm^{-1}): 2957(w), 2924(w), 1635(s), 1440(sh),1384(s), 1327(sh), 1268(sh), 1214(sh), 1091(s), 1025(w), 1004(w), 955(s), 919(s), 876(m), 799(s, br), 732(m), 591(s, br), 514(sh), 424(w).

4: $Na_{16}[(Mo_{12}O_{36}(HPO_3)_2(H_2O)_6)\subset Mo_{24}Fe_{12}(EDTA)_{12}O_{72}]\cdot 85H_2O$, Na_{16}**[4a]**$\cdot 85H_2O$, M.W.: 11478.35

Ethylenediaminetetraacetic acid disodium salt dihydrate (0.093 g, 0.25 mmol) and $FeCl_3\cdot 6H_2O$ (0.067 g, 0.25 mmol) were added to a solution of $Na_2MoO_4\cdot 2H_2O$ (0.180 g, 0.75 mmol) in water (8 mL). The mixture was vigorously stirred for 20 min until a clear red solution is formed. The solution was then acidified with 1 M HCl (0.3 mL) to pH 2.4, and H_3PO_3 (3.3 mg, 0.04 mmol) then added. After heating at 90 °C for 30 min, the clear yellow solution was allowed to cool to room temperature and kept in an open 50 mL Erlenmeyer flask undisturbed for 2 weeks. The resulting yellow diamond-like crystals were collected and dried in air (yield 60 mg, 31% based on Fe). Due to the photosensitivity of compound **4**, the dried sample was stored in dark. Elemental analysis, calc.: C, 12.45%; H, 2.94%; N, 2.90%; Na, 3.17%; P, 0.54%; Fe, 5.79%; Mo, 29.86%; found: C, 12.29%; H, 2.13%; N, 2.96%; Na, 2.83%; P, 0.56%; Fe, 5.58%; Mo, 29.30%. IR (KBr pellet,3000–400cm^{-1}): 2957(w), 2924(w), 1638(s), 1440(sh),1384(s), 1327(sh), 1268(sh), 1214(sh), 1109(sh), 1025(w), 1004(w), 950(s), 930(sh), 878(m), 789(m), 735(m), 599(s, br), 514(sh), 422(w).

4. Crystallographic data and crystal structures of 1-4

Table S1: Crystallographic Details for Compound **1-4**

Compound	**1**	**2**	**3**	**4**
Empirical formula	$C_{120}H_{310}Fe_{13}Mo_{24}N_{24}Na_{15}O_{272}$	$C_{120}H_{324}Fe_{12}Mo_{36}N_{24}Na_{15}O_{298}P$	$C_{120}H_{344}Fe_{12}Mo_{24}N_{24}Na_{18}O_{330}P_2W_{18}$	$C_{120}H_{328}Fe_{12}Mo_{36}N_{24}Na_{16}O_{301}P_2$
Formula weight	10391.00	11371.87	14161.99	11477.87
Temperature (K)	150(2)	150(2)	150(2)	150(2)
Crystal system	Cubic	Cubic	Cubic	Cubic
Space group	*F*d-3	*F*d-3	*F*d-3	*F*d-3
Unit Cell dimensions	a = 61.215(8) Å, α = 90 º	a = 60.6151 (8) Å, α = 90 º	a = 61.2120 (8) Å, α = 90 º	a = 61.0846 (8) Å, α = 90 º
	b = 61.215(8) Å, β = 90 º	b = 60.6151 (8) Å, β = 90 º	b = 61.2120 (8) Å, β = 90 º	b = 61.0846 (8) Å, β = 90 º
	c = 61.215(8) Å, γ = 90 º	c = 60.6151 (8) Å, γ = 90 º	c = 61.2120 (8) Å, γ = 90 º	c = 61.0846 (8) Å, γ = 90 º
Volume ($Å^3$)	229393(93)	222711(19)	229356 (20)	227927(13)
Z	16	16	16	16
Density (calculated) (Mg/m^3)	1.204	1.357	1.641	1.338
Absorption coefficient (mm^{-1})	1.032	1.177	4.495	1.154
F (000)	82192	89600	108032	90464
Reflections collected / unique	110685 / 9856 [R(int) = 0.1139]	143266 / 8349 [R(int) = 0.0770]	200908 / 11712 [R(int) = 0.1154]	104964 / 8340 [R(int) = 0.1074]
Data / restraints / parameters	9856 / 3 / 718	8349 / 9 / 728	11712 / 0 / 819	9856 / 3 / 642
Goodness-of-fit on F^2	1.214	2.210	1.1273	1.652
Final R indices [I>2δ (I)]	R1 = 0.0994, wR2 = 0.2484	R1 = 0.1483, wR2 = 0.3988	R1 = 0.0899, wR2 = 0.2064	R1 = 0.1227, wR2 = 0.3197
R indices (all data)	R1 = 0.1631, wR2 = 0.3512	R1 = 0.2179, wR2 = 0.5131	R1 = 0.1641, wR2 = 0.3038	R1 = 0.1967, wR2 = 0.4358
Largest diff. peak and hole (e. $Å^{-3}$)	1.08 and -0.63	2.21 and -1.39	1.66 and -1.09	2.33 and -0.70

Table S2. Bond valence sum (BVS) calculations of Mo and Fe atoms in **1–4**.

	**BVS (1)**	**BVS (2)***	**BVS (3)**	**BVS (4)**
Mo1	6.344	6.197	6.027	5.903
Mo2	6.240	6.093	6.136	6.173
Mo3	6.203	6.014	5.784	6.141
Mo4	6.051	6.156	5.989	6.444
Mo5	5.887	/	/	6.467
Mo6	/	/	/	5.920
Mo7	/	/	/	/
Mo8	/	/	/	/
Fe1	3.188	3.015	2.929	2.935
Fe2	2.842	2.959	3.090	2.998
Fe3	2.921	/	/	/

* The bond valence of Mo5-Mo8 on Keggin {PMo_{12}} could be calculated accurately due to the high disorder. Since no reducing species is introduced into the synthetic system, the BVS of Mo5-Mo8 could be therefore reasonably assigned as +6.

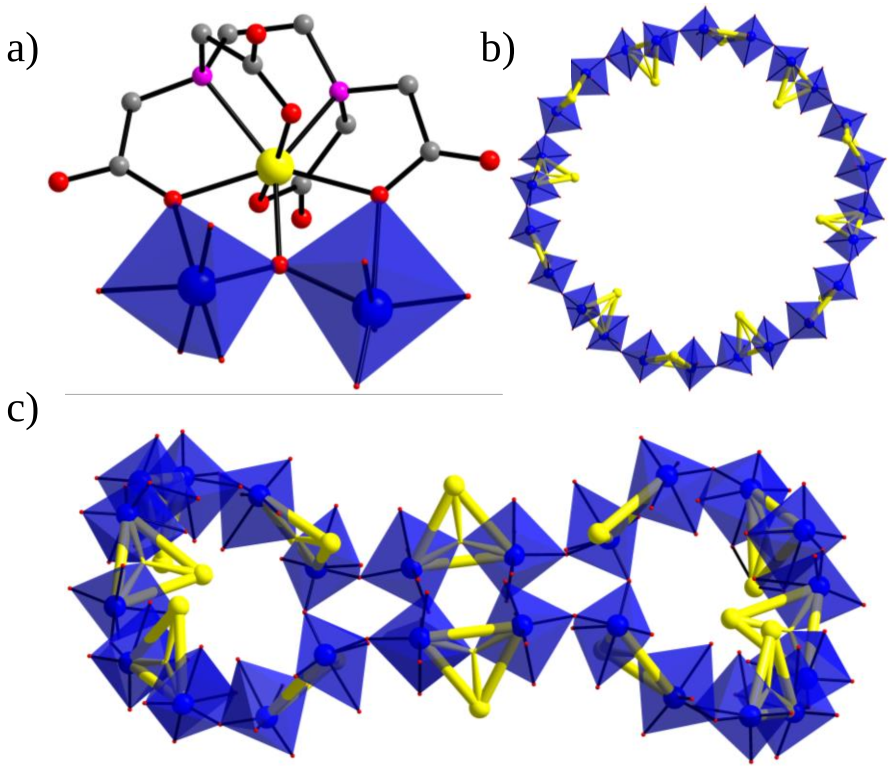

Figure S1. a) View of Mo_2Fe(EDTA) building unit; Top b) and side view c) of simplified Mo_{24} macrocycle with 12 Mo_2Fe triangels highlighted in yellow. EDTA ligands are omited for clarity.

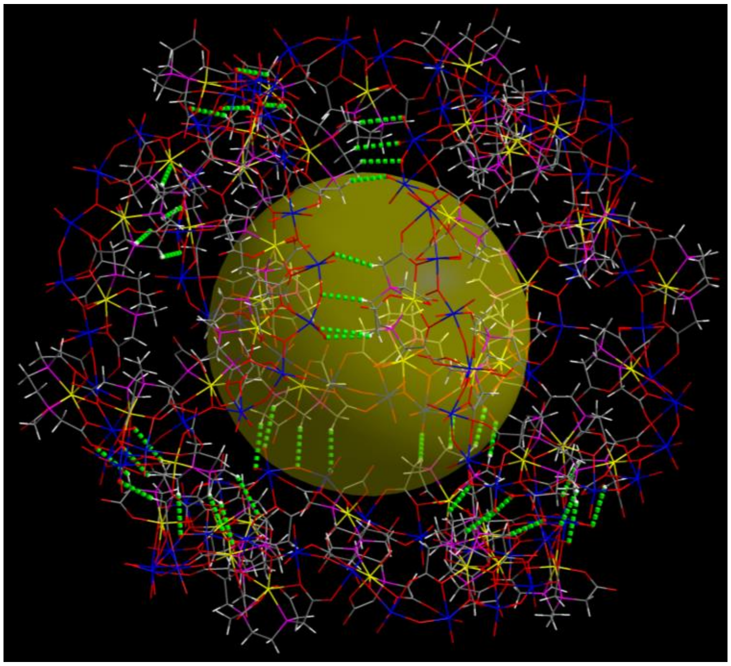

Figure S2. Hydrogen bonds fomed between terminal and bridging O atoms on Mo_{24} cycle and methyl groups on the glycine arms of EDTA that connect four **1a** macrocycles into one supramolecular tetrahedron.

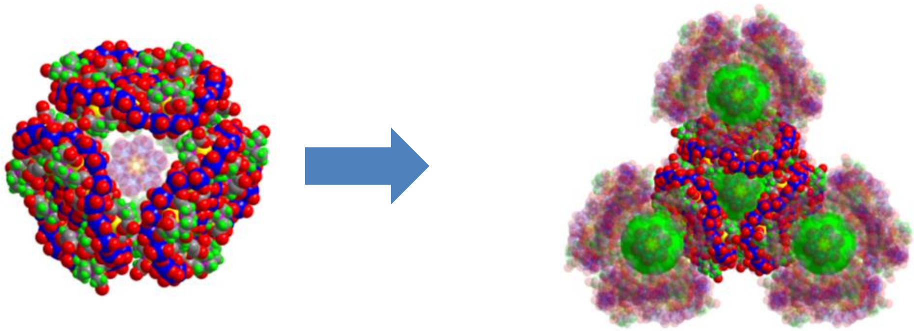

Figure S3. Space filling repsentaiton of tetrahedral arrange of one supramolecular tetrahedron with adjacent four tetrahedra by face-shared mode.

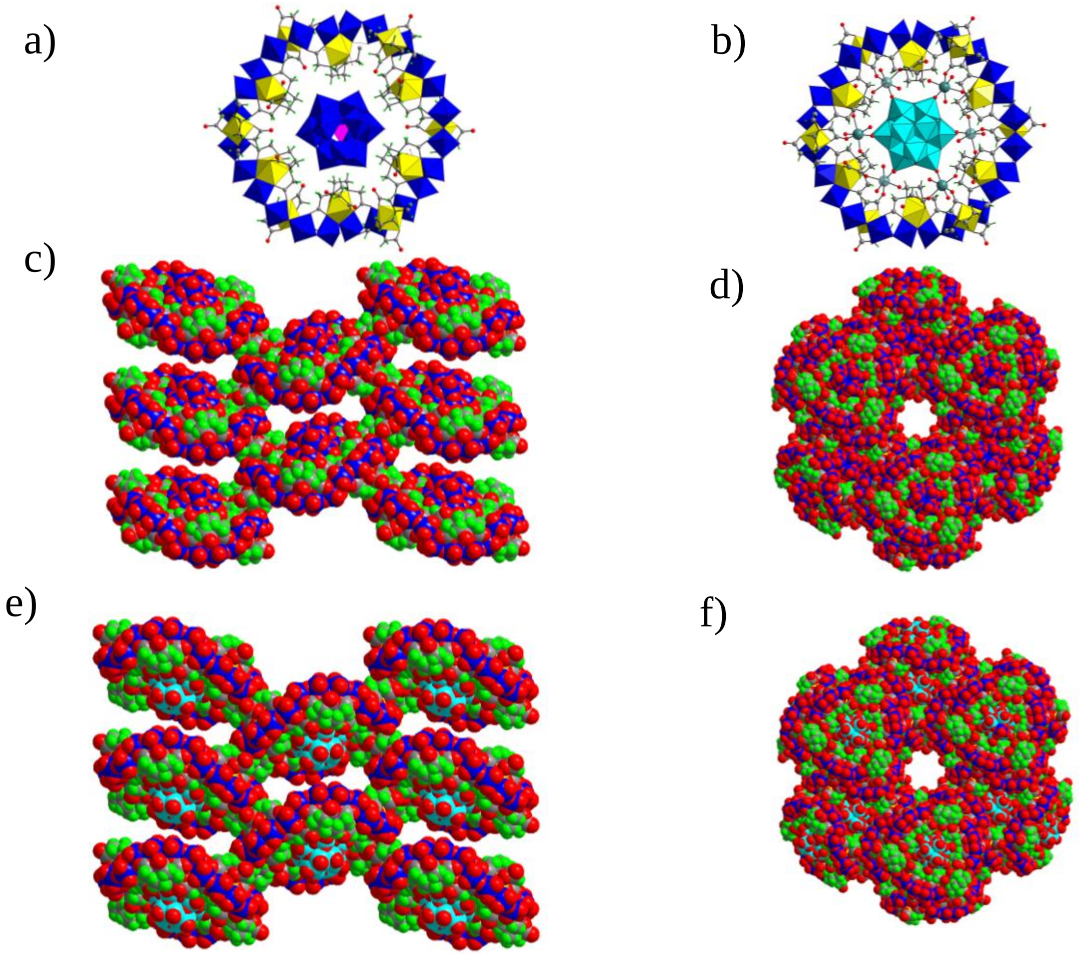

Figure S4. View of the molecular structure of **2a** a) and **3a** b). View of the different supramolecular structrures packed from **2a** (c) and $[(SiMo_{12}O_{40})\subset Mo_{24}Fe_{12}(EDTA)_{12}O_{72}]^{16-}$ (d), **3a** (e) and $[(P_2W_{18}O_{62})\subset Mo_{24}Fe_{12}(EDTA)_{12}O_{72}]^{16-}$ (f). Fe, yellow; Mo, blue; W, turquoise; Na, teal; P, pink ; O, red; C, gray; N, pink; H, bright green.

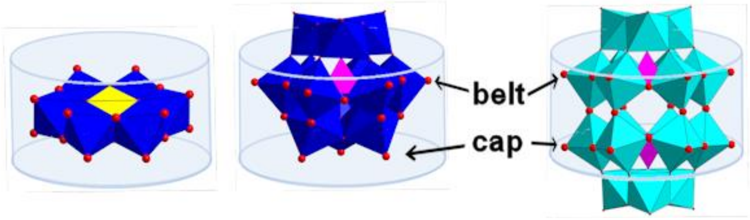

Figure S5. Schematic representation of surface oxygen atoms (red ball) on Anderson $\{FeMo_6\}$ (left), Keggin $\{PMo_{12}\}$ (middle) and Dawson $\{P_2W_{18}\}$ (right) guests involved in hydrogen bonds with macrocycle host (light blue cylinder).

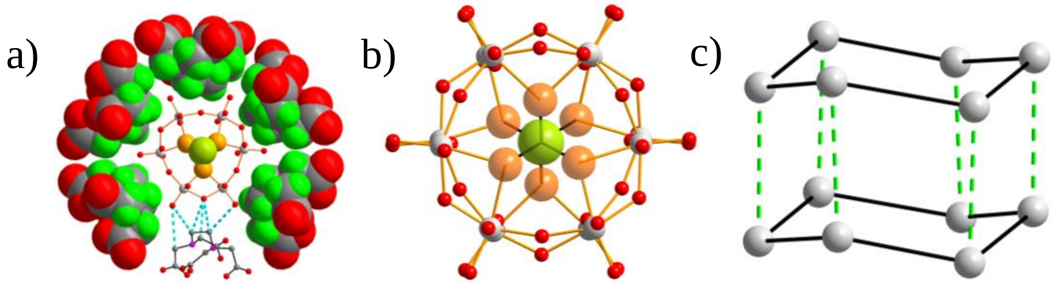

Figure S6. a) Representation of hydrogen bonds (turquoise dotted lines) formed between one sixth of $\{Mo_{12}(HPO_3)_2\}$ guest and one EDTA ligand in **4**; b) Top view of the $\{Mo_{12}(HPO_3)_2\}$; c) Representation of the chair conformation of two Mo_6 layers in $\{Mo_{12}(HPO_3)_2\}$, which are connected by green dash lines.

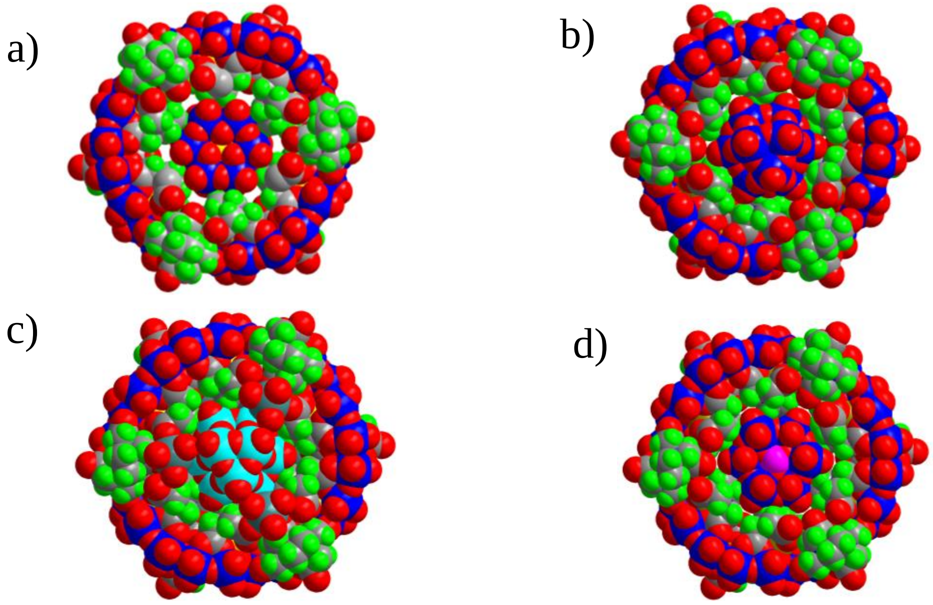

Figure S7. Space filling representation of a) **1a**, $Na_{15}[(FeMo_6O_{24}H_6)\subset Mo_{24}Fe_{12}(EDTA)_{12}O_{72}]$; b) **2a**, $Na_{15}[(PMo_{12}O_{40})\subset Mo_{24}Fe_{12}(EDTA)_{12}O_{72}]$; c) **3a**, $Na_{18}[(P_2W_{18}O_{62})\subset Mo_{24}Fe_{12}(EDTA)_{12}O_{72}]$; d) **4a**, $Na_{16}[(Mo_{12}O_{36}(HPO_3)_2(H_2O)_6)\subset Mo_{24}Fe_{12}(EDTA)_{12}O_{72}]$.

5. TGA results of compounds 1-4

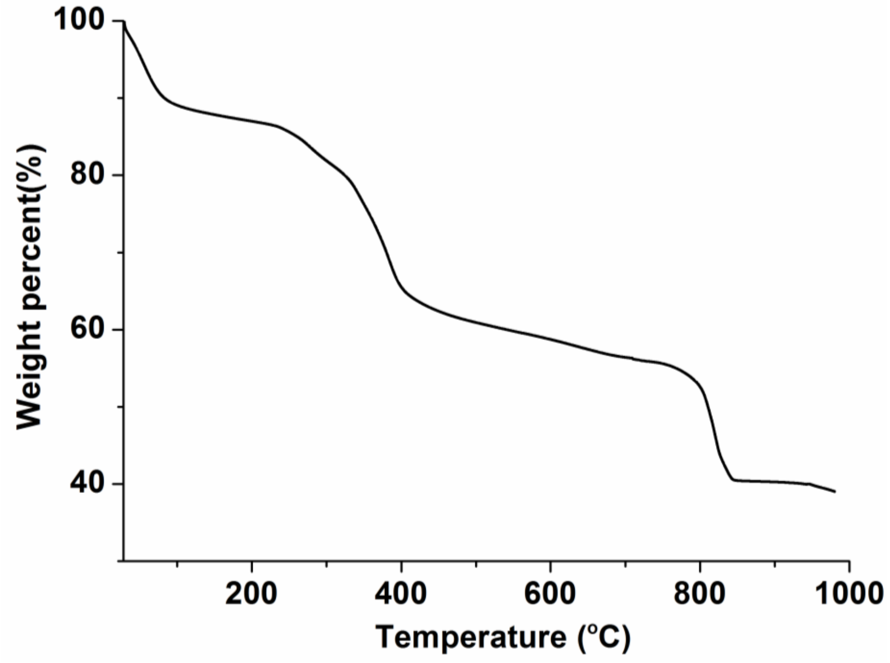

Figure S8. TGA curve for compound **1**. 13.2% weight loss from r.t. to 200 °C corresponds to ~80 H_2O.

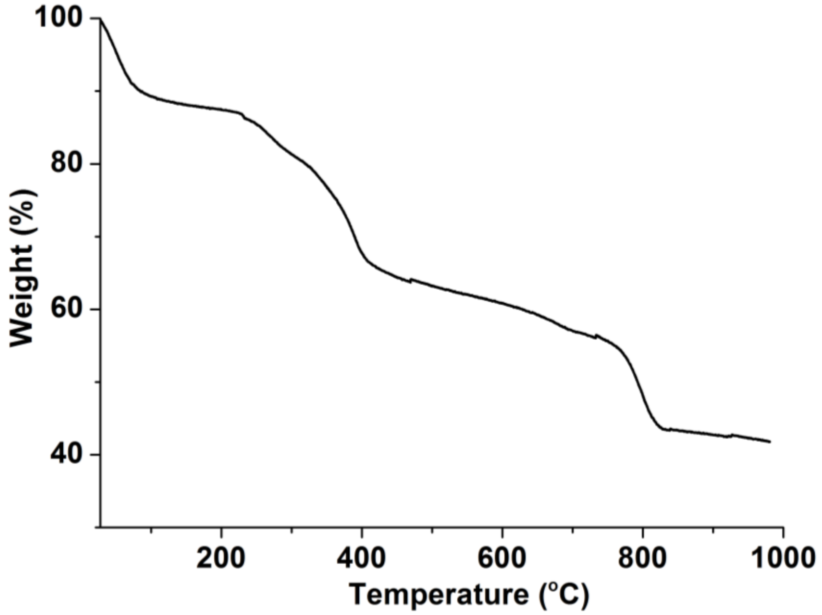

Figure S9. TGA curve for compound **2**. 13.5% weight loss from r.t. to 200 °C corresponds to ~90 H_2O.

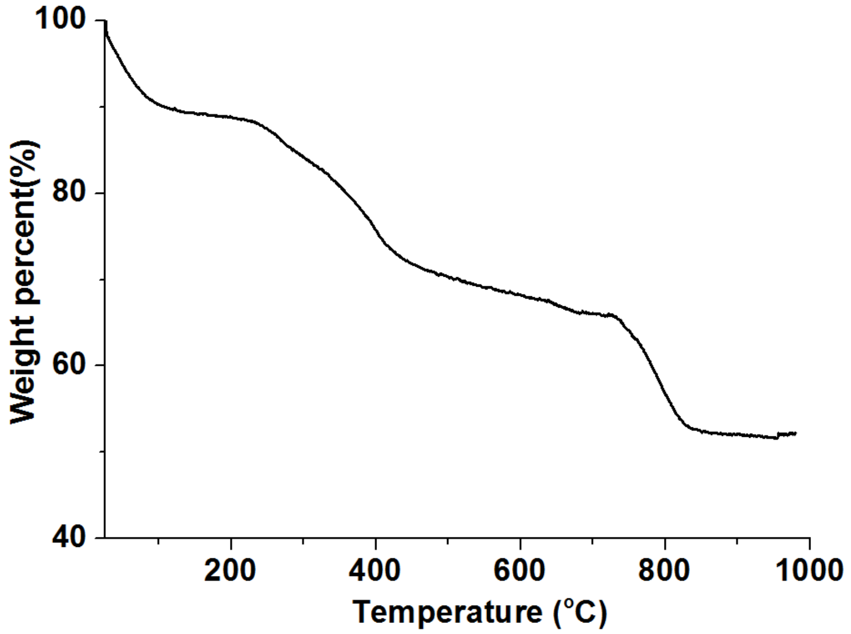

Figure S10. TGA curve for compound **3**. 12.9% weight loss from r.t. to 200 °C corresponds to ~100 H_2O.

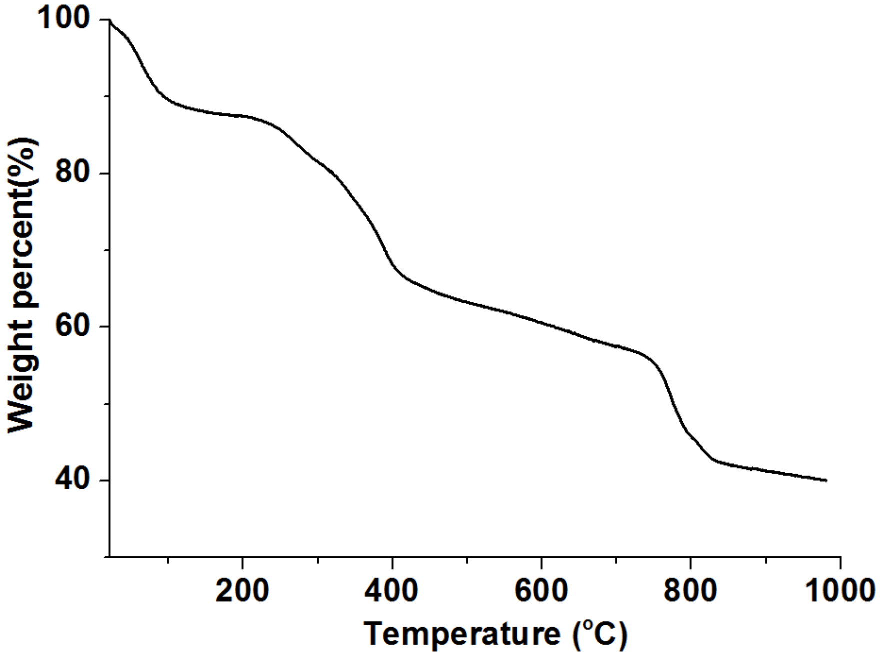

Figure S11. TGA curve for compound **4**. 13.3% weight loss from r.t. to 200 °C corresponds to ~85 H_2O.

6. PXRD spectra of 1

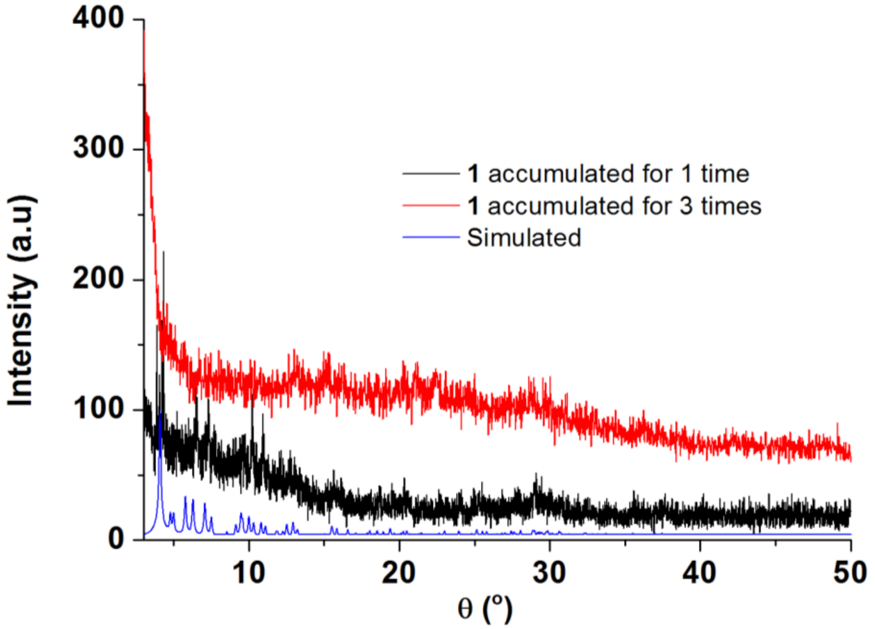

Figure S12. Experimental and simulated PXRD patterns of Compound **1**.

7. ESI-Ion Mobility Mass Spectrometry data of 1-4

Method:

IMS-MS spectroscopy samples were prepared by dissolving pure crystals of **1-4** in HPLC grade water, at approx. 5 mg/ml; these solutions were filtered and analysed with no further purification. All spectra were acquired on a Waters Synapt G2 HDMS instrument, with samples infused into the standard ESI source at 5 µl/min using a Harvard syringe pump. The following parameters were used for acquisition of all spectra (unless otherwise stated): ESI capillary voltage, 2.7 kV; sample cone voltage, 30 V; extraction cone voltage, 4.0 V; source temperature, 80 ℃; desolvation temperature, 150 ℃; cone gas (N2) flow, 15 L/h; desolvation gas (N2) flow, 750 L/h; source gas flow, 0 mL/min; trap gas flow, 2 mL/min; helium cell gas flow, 180 mL/min; IMS gas flow, 90 mL/min; IMS wave velocity, 750 m/s; IMS wave height, 25 V. Data was acquired using MassLynx v4.1 and initially visualised using DriftScope v2.2. IMS-MS spectra were further processed using UniDec[6] to allow clear visualisation and produce mass distribution spectra (i.e. deconvoluted "neutral mass spectra"). Briefly, the data processing workflow ran as follows (i) raw data files were loaded into UniDec; (ii) some filtering/processing was carried out – primarily subtraction of a curved background and gaussian smoothing in the m/z domain and application of a 5-10% minimum intensity threshold; (iii) peak width and shape was assigned using the UniDec GUI's dedicated tool; (iv)

manual assignment of peak charge was made in most cases, where charge was clearly observable; (v) deconvolution was run, yielding ‘cube’ figures of the IMS-MS data, and deconvoluted “neutral .

IMS-MS Spectra

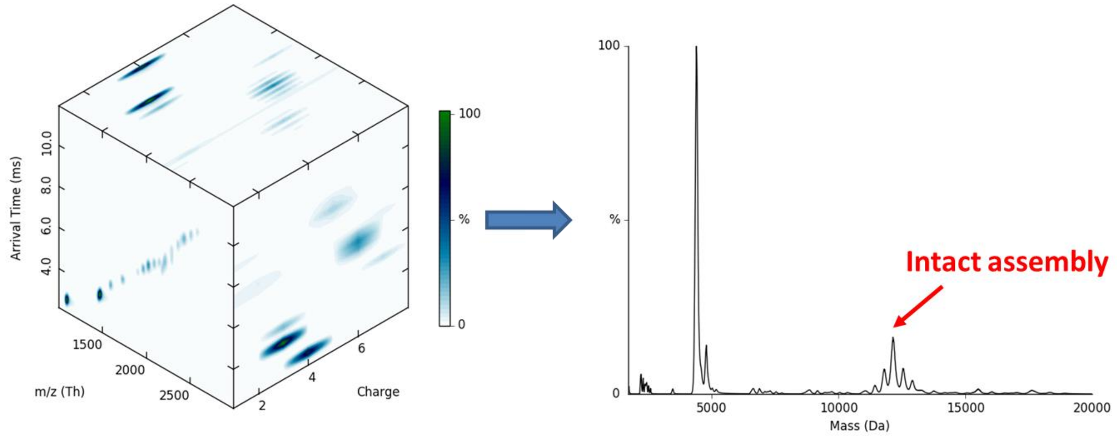

Fig S13. IMS-MS data (left) and deconvoluted neutral mass spectrum (right) of a solution of **3**.

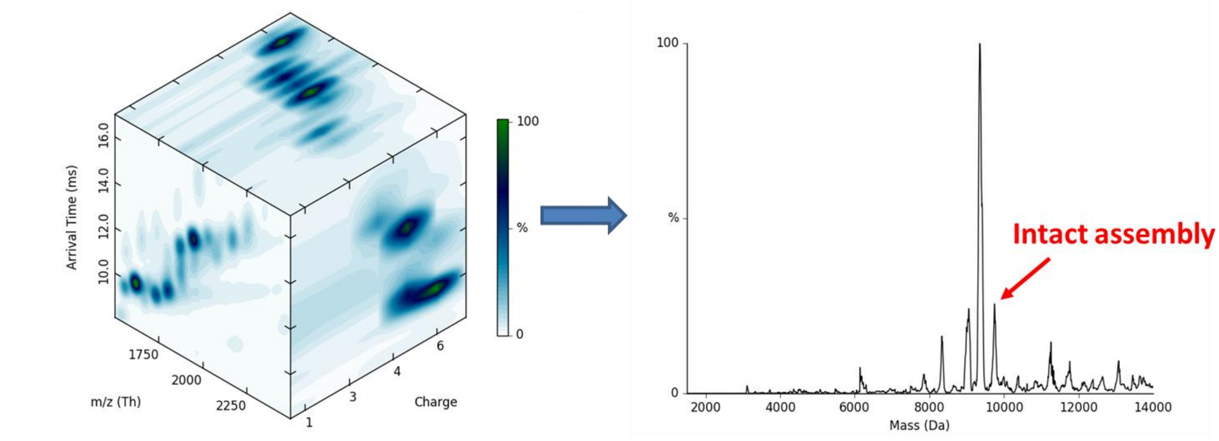

Fig S14. IMS-MS data (left) and deconvoluted neutral mass spectrum (right) of a solution of **4**.

8. Transformation from 1 to 2 and 3 by template exchange and ^{31}P NMR study of template exchange process

1. Transformation from 1 to 2 and 3 by template exchange

1 (0.11 g, 0.01 mmol) was dissovled in water (8 mL) by heating at 90 °C for 10 min to make a clear solution. Then $H_3PMo_{12}O_{40}$ (0.2 g, 0.1 mmol) or $Na_6P_2W_{18}O_{62}\cdot 14H_2O$ (0.475 g, 0.10 mmol) was added this solution and the pH value was adjusted to 2.4 by 1M NaOH or 1M HCl. After heating at 90 °C for 30 min, the clear yellow solution was allowed to cool to room temperature and kept in an open 50 mL Erlenmeyer flask undisturbed for 3 weeks. The resulting yellow diamond-like crystals were characterized by Singe-crystal X-ray diffraction and Elemental analysis, and identified as Keggin-templated **2** or Dawson-templated **3**.

2. ^{31}P NMR study of template exchange

$Na_6P_2W_{18}O_{62}\cdot 14H_2O$ (18 mg, 4.44 × 10^{-6} mol) was dissolved in 0.6 mL D_2O and the clear solution was used for ^{31}P NMR. Then **1** (16 mg, 1.48 × 10^{-6} mol) was added to the solution and heated at 90 °C for 10 min, ^{31}P NMR spectrum was recorded after the solution cooled to r.t. This process was repeated for three times until the ratio of $Na_6P_2W_{18}O_{62}\cdot 14H_2O$: **1** reached to 1:1. Note that: All the ^{31}P NMR spectra were obtained under the same conditions to make a comparison.

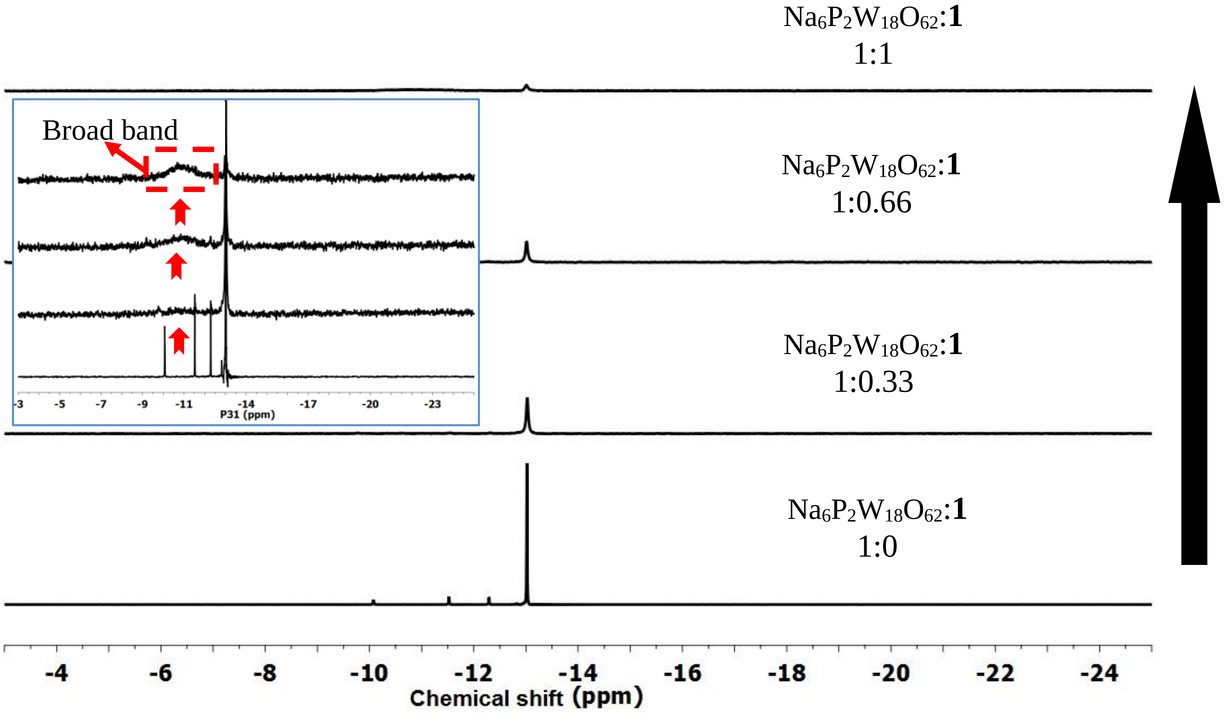

Figure S15. ^{31}P NMR spectral study of template exchange of **1** with $Na_6P_2W_{18}O_{62}$ (Dawson). From bottom to up: $Na_6P_2W_{18}O_{62}$:**1**, 1:0; $Na_6P_2W_{18}O_{62}$:**1**, 1:0.33; $Na_6P_2W_{18}O_{62}$:**1**, 1:0.66; $Na_6P_2W_{18}O_{62}$:**1**, 1:1. The intensity scale of four spectra is the same. The expanded spectra on top

left indicates the gradual integration of the peaks corresponding to minor impurities Preyssler (-10 ppm) and β-{P_2W_{18}} (-11.5 and -12.3 ppm) into a broad band.

9. BET plot and N_2 adsorption of 1

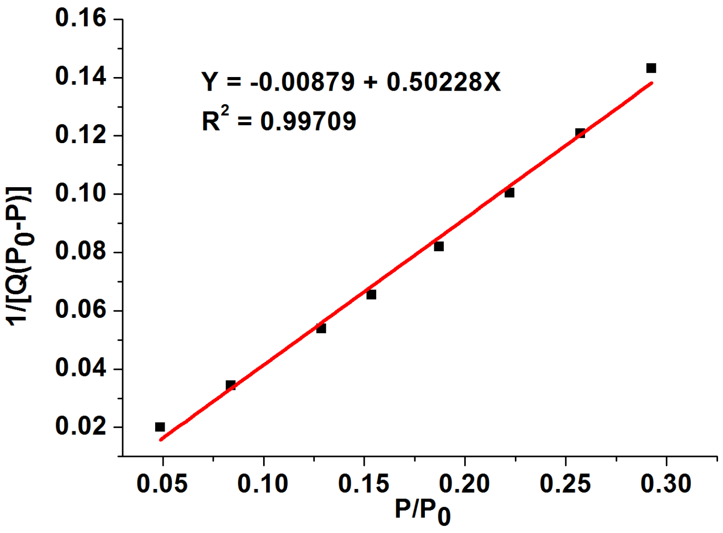

Figure S16. BET plot of **1**.

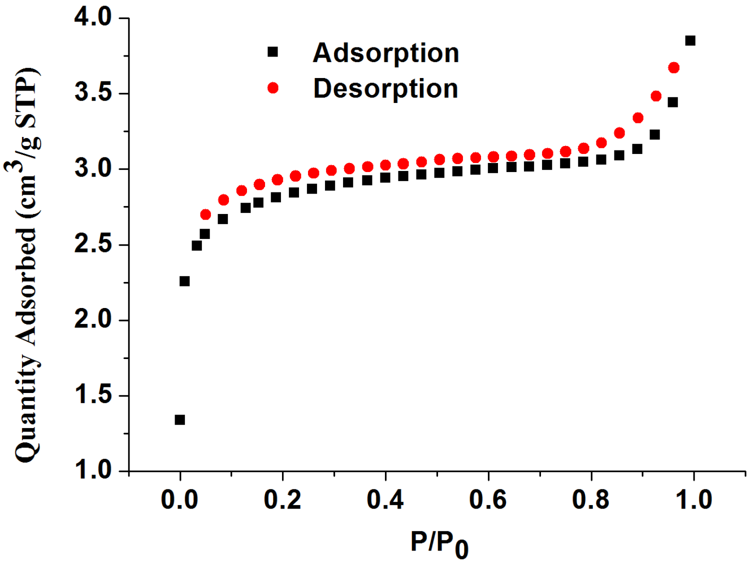

Figure S17. N_2 adsorption of **1**.

10. References

1. a) Lyon, D. K.; Miller, W. K.; Novet, T.; Domaille, P. J.; Evitt, E.; Johnson, D. C.; Finke, R. G. *J. Am. Chem. Soc.* **1991**, *113*, 7209–7221; b) Finke, R. G.; Droege, M. W.; Domaille, P. J.; *Inorg. Chem.* **1987,** *26***,** 3886-3896.

2. G. Sheldrick, *ActaCrystallographica Section A*, **1990**, *46*, 467-473.

3. G. Sheldrick, *ActaCrystallographica Section A*, **2008**, *64*, 112-122.

4. L. Farrugia, *J. Appl. Crystallogr*., **1999**, *32*, 837-838.

5. R. C. Clark, J. S. Reid, *ActaCrystallogr., Sect. A*,**1995**, *51*, 887-897.

6. M. T. Marty, A. J. Baldwin, E. G. Marklund, G. K. a. Hochberg, J. L. Benesch and C. V. Robinson, *Anal. Chem.*, **2015**, *87*, 4370-4376.
